# DKWM-XLSTM: A Carbon Trading Price Prediction Model Considering Multiple Influencing Factors

**DOI:** 10.3390/e27080817

**Published:** 2025-07-31

**Authors:** Yunlong Yu, Xuan Song, Guoxiong Zhou, Lingxi Liu, Meixi Pan, Tianrui Zhao

**Affiliations:** School of Economics & Management, Central South University of Forestry and Technology, Changsha 410004, China; 20225793@csuft.edu.cn (Y.Y.); liulx2022@csuft.edu.cn (L.L.); 20223881@csuft.edu.cn (M.P.); 20223799@csuft.edu.cn (T.Z.)

**Keywords:** carbon trading price prediction, decomposition framework, Kolmogorov–Arnold network, wavelet multi-head attention, XLSTM model, carbon trading market, multi-scale feature analysis, forestry carbon sinks, remote sensing

## Abstract

Forestry carbon sinks play a crucial role in mitigating climate change and protecting ecosystems, significantly contributing to the development of carbon trading systems. Remote sensing technology has become increasingly important for monitoring carbon sinks, as it allows for precise measurement of carbon storage and ecological changes, which are vital for forecasting carbon prices. Carbon prices fluctuate due to the interaction of various factors, exhibiting non-stationary characteristics and inherent uncertainties, making accurate predictions particularly challenging. To address these complexities, this study proposes a method for predicting carbon trading prices influenced by multiple factors. We introduce a Decomposition (DECOMP) module that separates carbon price data and its influencing factors into trend and cyclical components. To manage non-stationarity, we propose the KAN with Multi-Domain Diffusion (KAN-MD) module, which efficiently extracts relevant features. Furthermore, a Wave-MH attention module, based on wavelet transformation, is introduced to minimize interference from uncertainties, thereby enhancing the robustness of the model. Empirical research using data from the Hubei carbon trading market demonstrates that our model achieves superior predictive accuracy and resilience to fluctuations compared to other benchmark methods, with an MSE of 0.204% and an MAE of 0.0277. These results provide reliable support for pricing carbon financial derivatives and managing associated risks.

## 1. Introduction

As the world’s largest carbon market, China’s carbon pricing mechanism is of significant importance to climate economics research [1]. However, the market currently faces two major challenges: First, during the pilot phase, carbon trading prices suffer from insufficient liquidity and considerable price volatility, which can undermine the effectiveness of policy implementation [2]. Second, the role of carbon trading prices in driving industrial upgrading and the role of technological innovation have not been sufficiently emphasized [3]. According to our research, carbon trading prices not only reflect the dynamics of supply and demand but are also intricately linked to green technology innovation and environmental monitoring data derived from remote sensing technologies [4]. To guide policy-making and corporate decision-making [5], establishing a predictive model that incorporates both policy and industry data can provide more robust insights into the patterns of carbon trading price fluctuations [6]. This paper aims to facilitate the transition of the carbon market from quantity-based management to price regulation, thereby enhancing the alignment of carbon market efficiency with climate governance objectives [7]. Due to the influence of multi-factor coupling effects, carbon prices often exhibit characteristics such as uncertainty, non-stationarity, and high levels of noise, which present significant challenges for accurate carbon price forecasting. Given the influence of multi-factor coupling effects, which result in carbon prices exhibiting characteristics such as uncertainty, non-stationarity, and high noise [8], the development of an effective carbon price forecasting model is of paramount importance. Addressing these challenges is critical for improving the accuracy and reliability of carbon price predictions [9].

Carbon price forecasting fundamentally constitutes a time series modeling task [10]. Traditional statistical models are well-suited for data with small sample sizes and low volatility, as they capture underlying trends to facilitate predictions [11]. However, as carbon trading continues to evolve in China, the volume of carbon trading data has significantly increased [12], leading to a decline in the predictive performance of these traditional statistical models [13]. Recent advancements in artificial intelligence have introduced novel solutions and perspectives for overcoming the limitations of traditional statistical models [14]. Due to their ability to handle nonlinear relationships and automate feature extraction, machine learning and deep learning models have been increasingly applied to the prediction of carbon emissions trading prices.

The LSTM-CNN model proposed by Shi et al. (2024) improved training speed by 40% and enhanced prediction accuracy [15]. However, its adaptability in complex scenarios was not clearly defined. Huang et al. (2024) utilized Generative Adversarial Networks (GANs) to effectively extract time series features of carbon trading prices, with the iterative training of the generator and discriminator yielding accurate predictions [16]. However, the training process remains complex and computationally intensive. Yin et al. (2025) optimized the hyperparameters of the Transformer model using orthogonal matrix tuning and quantized loss functions, leading to improved prediction accuracy. Nevertheless, the complexity involved in hyperparameter tuning may limit the model’s practical applicability [17]. Lu et al. (2020) employed six models, including particle swarm optimization and support vector machines, for carbon price prediction. While this approach demonstrated the potential of optimization algorithms, the complexity of the models and the challenges associated with parameter tuning may impede their practical implementation [18]. The results of Kumar et al. (2024) demonstrate that the Random Forest and Gradient Boosting models significantly outperform the traditional ARIMA model [19]. While modern methods excel at capturing market dynamics, their black-box nature and interpretability issues present notable drawbacks. Consequently, the ET-MVMD-LSTM framework developed by Zhang et al. (2023), which integrates extreme random trees and multivariate variational modal decomposition to enhance data feature extraction, also introduces increased model complexity and higher computational costs, thereby complicating its practical application [20].

Although significant progress has been made in the field of carbon price forecasting, several challenges remain. In practical applications, carbon price forecasting faces three major issues:

(1) Carbon trading prices are influenced by the combined effects of multiple factors, including the macroeconomic environment, fluctuations in energy prices, and cyclical changes. The interaction of these factors results in high volatility and uncertainty in carbon emission prices, thereby complicating data analysis and modeling.
(2) The non-stationary volatility characteristics of carbon trading prices significantly increase the complexity of extracting time-series features. The inherent information redundancy within the data further diminishes the distinguishability of key features, making it challenging to effectively separate noise from valid signals. This, in turn, reduces the model’s accuracy in representing market dynamics and undermines its predictive robustness.
(3) Carbon trading prices are susceptible to unforeseeable factors, such as global health crises and geopolitical conflicts. For instance, the economic lockdowns induced by the COVID-19 pandemic led to a reduction in industrial production and demand for carbon emission rights, resulting in a decline in carbon prices, with this introduced additional uncertainty complicating forecasting efforts. Models may become overly sensitive to local noise while insufficiently capturing global trends, ultimately affecting the accuracy and reliability of the forecast results.

To address the challenge of high computational complexity in carbon price models arising from the coupled effects of multiple factors, Li et al. (2023) proposed a method that decomposes carbon trading price data in real time using a combination of Multiple Ensemble Patch Transformation (MEPT) and an improved Adaptive Noise–Complete Ensemble Empirical Mode Decomposition (ICEEMDAN) [21]. This approach captures trends and volatility patterns across multiple resolutions. By incorporating influencing factors from diverse domains, such as energy and stock markets, and employing Causal Time Convolutional Networks (CTCNs) for precise prediction and causal analysis, the model comprehensively accounts for the impact of multiple factors on carbon prices, thereby enhancing prediction accuracy. However, the high computational complexity and relatively intricate online deployment of this method limit its flexibility and practical applicability. Yang et al. (2024) proposed a novel Multi-Scale Interval Value Decomposition Integration (MIDE) module, which combines Noise-Assisted Multivariate Empirical Mode Decomposition (NAMEMD), Interval Value Vector Autoregression (IVAR) models, Interval Event Analysis (IEA), and Interval Multi-Layer Perceptron (IMLP) to predict EU carbon future prices [22]. This approach incorporates interval variables, such as Brent crude oil futures prices and the US Dollar Index. While the module offers certain advantages in accounting for market impacts on carbon prices, it heavily relies on interval variables, potentially limiting the model’s flexibility. The relatively high computational burden hinders practical application, necessitating the development of a more refined data decomposition module. This module should effectively reduce the model’s computational complexity under the influence of multi-factor coupling effects, while decreasing reliance on interval variables, thereby improving the accuracy of data decomposition.

To address the feature extraction challenges posed by carbon price fluctuations, Zhang et al. (2023) proposed a two-stage feature extraction module [23]. The module first employs Complete Ensemble Empirical Mode Decomposition with Adaptive Noise (CEEMDAN) for preliminary decomposition, followed by fuzzy entropy for feature screening. Secondary decomposition is then performed using Variational Mode Decomposition (VMD), combined with an adaptive reorganization strategy. This approach enhances extraction efficiency and reduces model complexity while quantifying time series complexity through fuzzy entropy. It performs secondary VMD decomposition on high-complexity subsequences and reorganizes these subsequences into components, including low-frequency trends and high-frequency fluctuations, based on a similarity complexity threshold. However, the superposition of dual decompositions may introduce redundant subsequences, and even with reorganization, there is a risk of noise amplification. Wang et al. (2023) propose a feature extraction module that integrates Extreme Gradient Boosting (XGBoost) and Partial Autocorrelation Function (PACF), effectively combining multi-period features [24]. The module uses XGBoost to filter important external variables and captures the historical correlation of carbon price series with PACF, thereby enhancing forecasting performance and model interpretability. However, XGBoost is sensitive to hyperparameter settings and incurs a higher computational cost, while PACF relies on the stationarity of the series, limiting its effectiveness with non-stationary or abrupt data. Overall, despite the various innovations in feature extraction presented by existing models, they continue to face challenges related to computational complexity and a high dependency on data quality. Therefore, it is crucial to design an optimized feature extraction module that minimizes the interference of redundant information while preventing performance degradation due to excessive computational complexity. The objective is to enhance the robustness and efficiency of the feature extraction process.

To address the challenges of overfitting and underfitting in carbon trading models, Wang et al. (2023) propose a dual attention mechanism, consisting of a feature attention-based encoder and a temporal attention-based decoder [25]. This mechanism effectively mitigates uncertainty in carbon trading prices by dynamically capturing nonlinear features and temporal dependencies. Although the dual-attention mechanism improves prediction accuracy, the model’s high computational complexity, heavy reliance on data quality and preprocessing, and limited interpretability constrain its practical application. Ren et al. (2025) introduce the multi-head self-attention mechanism, which transforms the input sequence into Query, Key, and Value vectors through linear transformations [26]. By calculating and normalizing attention weights using Softmax, this mechanism dynamically allocates weights to emphasize key information, thereby enhancing the model’s ability to extract global features and improving prediction accuracy. However, the introduction of multi-head structures and multiple modules significantly increases the model’s overall complexity, resulting in high computational resource consumption, prolonged training times, and an elevated risk of overfitting due to the excessive number of parameters. Consequently, the use of algorithms such as Bayesian optimization is required to adjust hyperparameters and balance performance with efficiency. In summary, while existing attention mechanisms have made significant advancements in addressing market dynamics, they continue to face challenges related to high computational complexity and increased risks of overfitting in large-scale sequence modeling. To balance model performance, regularization techniques or structural simplifications are essential. Consequently, there is a need to propose an efficient attention mechanism that can mitigate the issues of computational complexity and overfitting, while reducing resource consumption and improving predictive accuracy and response speed, especially when dealing with uncertain events.

Despite advancements made by the aforementioned methods, challenges such as the coupling of multiple influencing factors, interference from redundant information, and sporadic uncertainty of events continue to threaten model performance. This paper, therefore, presents the following main contributions:

(1) We examine the impact of the coupled effects of multiple factors on carbon trading price prediction. Initially, all features and target variables are normalized using MinMaxScaler to mitigate differences in magnitude. Subsequently, the feature data is reshaped into a 3D tensor structure to meet the input requirements of the (eXtended Long Short-Term Memory) XLSTM network. This preprocessing approach not only effectively integrates various external factors influencing carbon trading prices but also ensures that the data is learned on a uniform scale, thereby enhancing the accuracy and stability of carbon price predictions. This aspect has been scarcely addressed in previous hybrid models.
(2) We introduce a novel DKWM-XLSTM model (Enhancing XLSTM with Decomposition, KAN-MD, and Wave-MH Attention Mechanisms), which incorporates three innovative features designed to improve the model’s performance and stability.
  (a) We propose a novel Decomposition (DECOMP) module designed to decompose input time series data into two components: cyclical and trend. The cyclical component captures short-term fluctuations, while the trend component reveals long-term changes. Within the XLSTM network, the sLSTM Block focuses on the cyclical component, while the mLSTM Block addresses the trend component. This decomposition method enhances the robustness of time series forecasting and is integrated with module-specific processing in the carbon price forecasting model. This approach effectively mitigates the influence of multiple factors.
  (b) We propose a novel Kolmogorov–Arnold Network with Multi-Domain Diffusion (KAN-MD) module, which integrates with the sLSTM Block to form the new sKAN module. Its adaptive univariate function retains only the nonlinear dependencies between key factors, while suppressing the ineffective coupling of secondary factors, thereby dynamically eliminating redundant information. Furthermore, adjusting function parameters in an interpretable manner directly identifies the core driving factors, significantly enhancing the accuracy of feature extraction in the carbon price prediction model.
  (c) We propose a novel Wave-Multi-Head Attention (Wave-MH attention) module, which integrates with the mLSTM Block to form the mWM module. The wavelet transform decomposes time series data into various frequency components, while the integration with the Multi-Head attention mechanism enables the model to focus on multiple dimensions of the input data and learn the relationships between different features. This combination allows the attention mechanism to concentrate on multi-scale features, thereby improving the model’s ability to mitigate the risks of overfitting and underfitting in carbon price prediction.

The DKWM-XLSTM model proposed in this study effectively extracts the trend and cyclical features of carbon trading prices from the Hubei Carbon Emission Rights Exchange, thereby enhancing the model’s robustness and enabling it to adapt more flexibly to dynamic data changes. The model demonstrates exceptional performance in evaluation metrics such as MSE, MAPE, MAE, and R^2^, with values of 0.204%, 0.0277, 9.25, and 96.06%, respectively. It is capable of predicting carbon trading prices with both speed and accuracy, offering valuable insights for the application of deep learning techniques in carbon trading price forecasting.

## 2. Materials and Methods

### 2.1. Data Acquisition and Processing

In this section, we employ ‘*Stata 17*’ to analyze carbon market data from Hubei Province, spanning from January 2017 to October 2024, alongside several influencing factors. The dataset was divided into 80% for training and 20% for testing, with the validity of the data confirmed through descriptive statistical analysis. Correlation analysis reveals a significant linear relationship between GDP and carbon trading prices, highlighting a multidimensional dynamic interaction. Following the Augmented Dickey–Fuller (ADF) test [27], all variables demonstrated strong stationarity after first-order differencing. The core variable, lnY, representing the carbon price, also exhibited inherent stationarity, establishing a solid foundation for the subsequent model construction. Additionally, the Granger Causality Test [28] uncovers the complexities underlying the carbon price formation mechanism. These analytical results affirm the robustness and applicability of the predictive model.

Our dataset encompasses carbon market data from Hubei Province, spanning from January 2017 to October 2024, along with several factors influencing carbon trading prices, including liquefied natural gas prices, gasoline prices, diesel prices [29], as well as China’s inflation rate, CPI, PPI, manufacturing PMI, and GDP, as detailed in Table 1. All data was sourced from the WIND database. For model construction, we utilized the training set for model development and the test set for performance evaluation. Given the limited data, a 90% training set partition proved insufficient for validating the model’s effectiveness, prompting us to explore 80% and 85% training–test splits. Ultimately, we allocated 80% of the data to the training set and 20% to the test set. The training set comprises data from 1482 trading days, covering the period from 3 January 2017 to 7 August 2022, while the test set includes data from 370 trading days, spanning from 8 August 2023 to 10 October 2024.

This section outlines the characteristics of the variables, presenting the sample size, mean, standard deviation, minimum, median, and maximum values, as detailed in Table 2. Specifically, we applied logarithmic transformation to the carbon price and the other eight influencing factors [30], except for the inflation rate, due to its relatively small values. The descriptive statistics reveal that the statistical properties of the variables are within reasonable ranges, underscoring the rigor and validity of the dataset.

Additionally, we perform a Correlation Analysis using both Pearson and Spearman coefficients [31] to assess the relationships between variables, with the results presented in Table 3. The Pearson coefficients are displayed in the lower left corner, while the Spearman coefficients are shown in the upper right corner. The analysis reveals that the selected indicator system effectively captures the triple interaction mechanism of cost–push, demand–pull, and policy regulation in the formation of carbon trading prices. A direct linear relationship is observed between GDP and carbon trading prices (r = 0.8167 ***), while nonlinear systemic effects arise from the energy–inflation–policy hedging chain. This is exemplified by the positive correlation between diesel prices and GDP, and the inverse relationship between PMI and CPI. This multidimensional dynamic correlation equips the model with time-series adaptability, spatial extensibility, and policy sensitivity, thereby providing a robust foundation for constructing time-varying parameter models and conducting stress tests under extreme scenarios. Consequently, it substantially improves the explanatory power and decision-support capacity of the carbon price forecasting model in the face of economic fluctuations and policy changes.

To mitigate the risk of “pseudo-regression” caused by non-stationary data, we first conducted the Augmented Dickey–Fuller (ADF) test on each variable, with the results presented in Table 4. Following rigorous unit root testing, all variables exhibited strong stationarity (|ADF| > 40) after first-order differencing, thereby providing a robust foundation for the subsequent construction of the Extended Long Short-Term Memory (XLSTM) model. Additionally, we performed Granger causality tests to further elucidate the multidimensional characteristics of the carbon price formation mechanism. The selection of variables comprehensively spans four key domains: economic pressure transmission, the role of policy regulation, fluctuations within the industrial chain, and shifts in the energy structure, thus offering a comprehensive depiction of the multilevel causal network underlying carbon price fluctuations.

Subsequently, we implement a systematic data preprocessing workflow to ensure the quality and suitability of the model input data. Data integrity is preserved through forced-type conversion and handling of missing values, while anomalous transaction records are filtered out based on business logic. Transaction dates are then decomposed into time-series features such as year, month, and day, and irrelevant categorical variables are removed. All features and target variables are normalized using MinMaxScaler to eliminate magnitude discrepancies [32], and the feature data is reshaped into a three-dimensional tensor structure (samples × time steps × features) to meet the input requirements of LSTM networks. Following this, the dataset is partitioned using a stratified sampling strategy (training set: test set = 8:2; 8.5:1.5). This preprocessing method enhances the training efficiency of the deep learning model by normalizing and transforming dimensions while preserving the temporal characteristics of the original data, thereby providing a robust data foundation for the accurate prediction of carbon emissions trading volume.

### 2.2. Method

**Figure 1:**
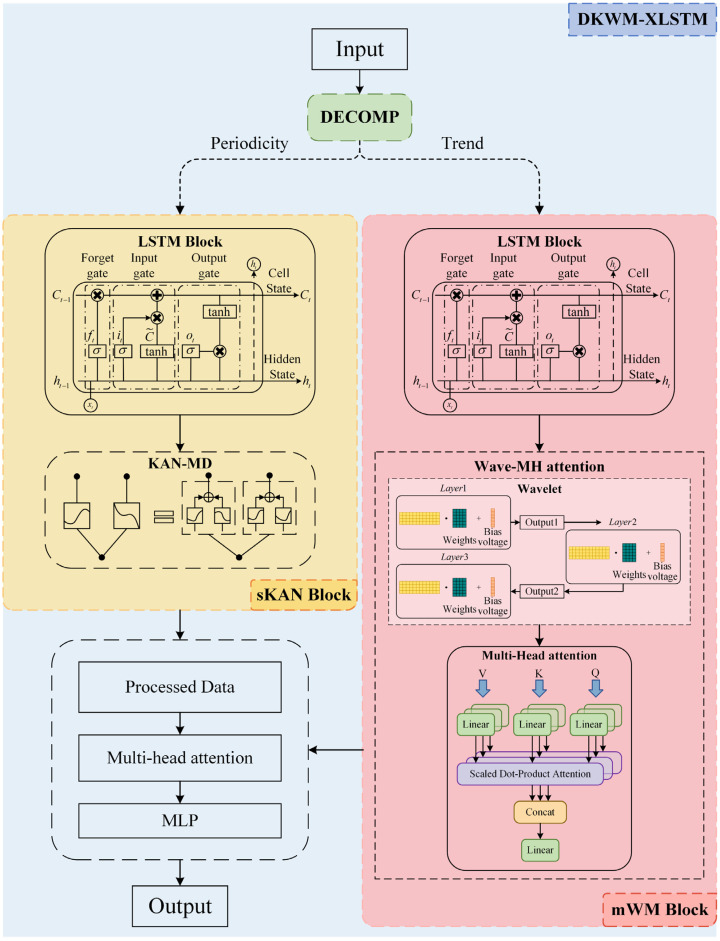
Architecture diagram of DKWM-XLSTM.

This section will introduce the model we adopted. Based on XLSTM, we have integrated three innovative components to enhance the predictive capability for carbon prices, which we have named DKWM-XLSTM. The specific components of this model include the following: Section 2.2.1 Decomposition (DECOMP), Section 2.2.2 Kolmogorov–Arnold Network with Multi-Domain Diffusion (KAN-MD), and Section 2.2.3 Wave-Multi Head Attention (Wave-MH Attention). Furthermore, the network diagram (Figure 1 below) illustrates the fundamental principles of the model design.

#### 2.2.1. Decomposition (DECOMP)

The Decomposition (DECOMP) module decomposes the carbon price into trend and cyclical components using a sliding average and cyclical adjustment method, which captures long-term economic changes and cyclical market fluctuations, respectively. The module dynamically adjusts the window size according to the length of the data, effectively mitigating the impact of random noise while preserving the underlying change patterns. The trend component reflects long-term influencing factors, such as industrial policies, while the cyclical component accounts for repetitive fluctuations, such as those associated with quota cycles. The decomposition results clearly differentiate the driving factors across various time scales, enabling the model to intuitively analyze the dynamic interactions between carbon trading prices and markets, including energy and commodities. This enhances the model’s adaptability to the complex interplay of multiple factors [33].

The DECOMP module is designed based on the additive model of time series decomposition, with its core theoretical framework decoupling the original signal $x_{t}$ into a trend term $T_{t}$ and a cyclical term $S_{t}$. The trend term $T_{t}$ reflects long-term economic changes, while the cyclical term $S_{t}$ captures market fluctuations. This decomposition process achieves trend extraction using a sliding average filter. The parameter m denotes the dynamic half-window width, and the window size is determined using an adaptive strategy. By default, a 7-point sliding average is applied when the sequence length exceeds 14, while one-third of the sequence length is used for shorter sequences to maintain a balance between filtering smoothness and signal fidelity. In the period term calculation stage, the algorithm initially computes the periodic component through a residual method, followed by periodicity correction to enhance feature stability. This correction process employs the Fast Fourier Transform to compute the autocorrelation function, and, after detecting the dominant cycle length *P*, performs a cycle folding averaging operation. Where *N* represents the number of complete cycles, this step effectively mitigates the impact of random noise on the periodic pattern.

(1) $$x_{t}=T_{t}+S_{t}$$

(2) $$T_{t}=\frac{1}{2m+1}\sum_{k=-m}^{m}x_{t+k}$$

(3) $$S_{t}=x_{t}-T_{t}$$

(4) $$\hat{S_{t}}=\frac{1}{N}\sum_{k=0}^{N-1}S_{t+kP}$$

After that, the decomposed data is added to the sKAN module and mWM module, respectively.

To begin with, the trend term $T_{t}$ is input to the mWM block to capture the long-term trend characteristics, and the mWM block is updated with the following equations:

(5) $$f_{t}=\sigma (W_{f}\cdot \left[h_{t-1},T_{t}\right]+b_{f})$$

(6) $$i_{t}=\sigma (W_{i}\cdot \left[h_{t-1},T_{t}\right]+b_{i})$$

(7) $$o_{t}=\sigma (W_{o}\cdot \left[h_{t-1},T_{t}\right]+b_{o})$$

(8) $$C_{t}=f_{t}\cdot C_{t-1}+i_{t}\cdot \tilde{C_{t}}$$

(9) $$h_{t}^{M}=o_{t}\cdot \tanh(C_{t})$$

The $f_{t}$, $i_{t}$, $o_{t}$ are the forgetting gate, input gate, and output gate, respectively. $C_{t}$ is the cell state, and $h_{t}^{M}$ is the hidden state of the mWM block.

Moreover, the period term $S_{t}$ is input to the sKAN block to capture the periodic fluctuations. The update equation of the sKAN block is similar to that of the mWM block:

(10) $${f^{\prime }}_{t}=\sigma ({W^{\prime }}_{f}\cdot \left[{h^{\prime }}_{t-1},S_{t}\right]+{b^{\prime }}_{f})$$

(11) $${i^{\prime }}_{t}=\sigma ({W^{\prime }}_{i}\cdot \left[{h^{\prime }}_{t-1},S_{t}\right]+{b^{\prime }}_{i})$$

(12) $${o^{\prime }}_{t}=\sigma ({W^{\prime }}_{o}\cdot \left[{h^{\prime }}_{t-1},S_{t}\right]+{b^{\prime }}_{o})$$

(13) $${C^{\prime }}_{t}={f^{\prime }}_{t}\cdot {C^{\prime }}_{t-1}+{i^{\prime }}_{t}\cdot {\tilde{C_{t}}}^{\prime }$$

(14) $$h_{t}^{S}={o^{\prime }}_{t}\cdot \tanh({C^{\prime }}_{t})$$

And the $h_{t}^{S}$ is the hidden state of the sKAN block.

The two channels process different features separately, and finally, their hidden states are combined to generate the prediction results. The integration of the output is achieved by splicing. The combined output is:

(15) $$h_{t}=concat(h_{t}^{M},h_{t}^{S})$$

Furthermore, the predicted values are obtained through the fully connected layer as follows:

(16) $$\hat{Y_{t}}=W_{p}\cdot h_{t}+b_{p}$$

where $\hat{Y_{t}}$ is the final prediction result, and $W_{p}$ and $b_{p}$ are the weights and biases of the fully connected layer, respectively.

We address the challenge of carbon prices being influenced by the coupled effects of multiple factors by innovatively integrating the DECOMP module with the Extended Long Short-Term Memory Network (XLSTM) [34]. A dual-channel structure is established by inputting the decomposed trend component into the mWM module and the periodic component into the sKAN module, effectively disentangling the dynamic characteristics of carbon prices across different time scales. The mWM module captures long-term trend changes, while the sKAN module focuses on periodic fluctuations, together providing an accurate representation of the carbon price patterns influenced by multiple coupled factors. Moreover, this clear division of labor not only enhances the model’s interpretability, offering an intuitive foundation for understanding the driving mechanisms behind carbon price changes, but also facilitates an effective integration of time series decomposition and deep temporal modeling under multi-factor coupling influences.

#### 2.2.2. Kolmogorov–Arnold Networks with Multi-Domain Diffusion (KAN-MD)

Traditional Multi-Layer Perceptrons (MLPs) learn features through the sequential connections of nonlinear processing across multiple layers [35]. However, their relatively simple network structure makes them vulnerable to noise when processing complex data, particularly in the analysis of data from physical systems with strong nonlinear relationships. Conventional parameter update methods often struggle to effectively distinguish between true signals and random fluctuations, which limits the model’s generalization ability. Inspired by the Kolmogorov–Arnold function representation theorem [36], we designed a compact feature interaction component called the KAN-MD module, with a parameter scale of 1.7 × 10^4^. This component is embedded between deep encoders and adaptively adjusts the coupling strength of cross-layer features, significantly improving the model’s ability to analyze complex correlated features.

The module employs an LSTM unit to extract temporal features and generate intermediate outputs. It then preprocesses the input tensor for subsequent linear processing. During forward propagation, the input data x is transformed by the learnable weights W and bias b of each $Z_{input}$ layer, yielding the outputs z and activated features of each layer $x_{c}$. Our KAN-MD module is divided into three layers. The activation function ReLU is applied, and batch normalization is used to mitigate the risk of overfitting. The specific formula is as follows:

(17) $$\begin{array}{c} Z_{input}=W_{input}x_{c}+b_{input} \end{array}$$

(18) ReLU(x)=Max(0,x)

(19) $$\begin{array}{c} x_{c}=ReLU\left(Z_{input}\right) \end{array}$$

where *W*, *b*, *c* represent the weights, biases, and number of layers, respectively, which are randomly initialized to small values before training begins. The KAN-MD module is a three-layer fully connected network consisting of two hidden layers with ReLU activation and a linear output layer. The input data undergoes two layers of nonlinear transformation and is then adjusted to the target dimension by the output layer. This structure implements feature dimension conversion and abstraction in a simplified manner while retaining the core idea of the Kolmogorov–Arnold theorem.

The loss function used is the Mean Squared Error (MSE), which quantifies the difference between the predicted values of the model and the true values. It is given by the following equation:

(20) $$J(\theta )=\frac{1}{N}\sum_{i=1}^{N}{\left(y_{true}^{\left(i\right)}-y_{pred}^{\left(i\right)}\right)}^{2}$$

where *N* is the number of samples, $y_{true}^{\left(i\right)}$ is the true value of the *i*-th sample, $y_{pred}^{\left(i\right)}$ is the predicted value of the *i*-th sample, and θ denotes the parameters of the model.

In the backpropagation stage, the model parameters are updated more efficiently using the AdamW optimizer [37], which combines adaptive learning rate adjustment with weight decay. The core principle of the gradient descent algorithm is to minimize the loss function by calculating the gradient of the loss function with respect to the model parameters and updating the parameters in the opposite direction of the gradient. The specific formula is as follows:

(21) $$\theta_{t+1}=\theta_{t}-\eta \cdot \nabla_{\theta }\cdot J(\theta )$$

where $\theta_{t}$ represents the model parameter at the *t*-th iteration, η is the learning rate, which controls the step size for parameter updates, and $\nabla_{\theta }J(\theta )$ is the gradient of the loss function J(θ) with respect to the parameter θ.

The innovation of the KAN-MD module lies in its ability to effectively simulate complex function mappings through multi-layer nonlinear transformations and learnable ReLU activation functions. This enhances the model’s adaptability to varying data distributions and tasks. The KAN-MD module improves parameter efficiency while effectively managing high-dimensional data and complex relationships. It significantly enhances the model’s capacity to capture and integrate multidimensional features of carbon prices, thereby improving the precision and efficiency of feature extraction. The data and information processed through the KAN-MD module enable the model to better integrate these multidimensional features, laying a solid foundation for the network’s overall performance improvement.

#### 2.2.3. Wave-Multi-Head Attention (Wave-MH Attention)

The integration of the wavelet transform and multi-head attention mechanism is primarily implemented in the mWM Block. The wavelet transform performs multi-scale decomposition on the input data [38], while the multi-head attention mechanism captures global dependencies within the data [39]. This fusion significantly enhances the accuracy of carbon price prediction by extracting multi-scale features and modeling global relationships.

Wavelet transform is used to perform multi-scale decomposition on input data. The purpose of wavelet transform is to decompose the input signal into wavelet coefficients of multiple scales, capturing the multi-scale features of the signal. This provides richer input features for subsequent multi-head attention mechanisms, thereby enhancing the model’s ability to model complex time series data. The wavelet transform decomposes the signal into coefficients across multiple scales, and combines with the characteristics of the dataset, as expressed in the following equation:

(22) $$W(a,b)=\int_{-\infty }^{\infty }f[n]\cdot \psi_{a,b}(t)\cdot dt$$

where f[n] is the input signal, $\psi_{a,b}(t)$ is the wavelet basis function, *a* is the scale parameter, and *b* is the shift parameter. Wavelet transforms are mainly divided into Continuous Wavelet Transforms (CWTs) and Discrete Wavelet Transforms (DWTs). Doroshenko et al. (2025) used CWT combined with Morlet wavelet basis functions to analyze the futures prices of three key commodities: palladium, heating oil, and copper [40].

By comparing different wavelet basis functions, we selected the relatively stable Haar wavelet basis function [41] to decompose the signal. The experimental results are shown in Table 5.

The Haar wavelet consists of two basic functions. One is the scale function used to generate the approximation coefficient cA[k], which extracts the low-frequency trend of the signal. It is defined as follows:

(23) $$\phi (t)=\left\{\begin{array}{l} 1,if0\leq t\leq 1\\ 0,otherwise \end{array}\right.$$

In addition, the corresponding low-pass filter is h[n]:

(24) $$h[n]=\left[\frac{1}{\sqrt{2}},\frac{1}{\sqrt{2}}\right]$$

Another is the wavelet function, which is used to generate the detail coefficient cD[k] and extract high-frequency fluctuations in the signal. It is defined as follows:

(25) $$\varphi (t)=\left\{\begin{array}{l} 1,if0\leq t<0.5\\ -1,if0.5\leq t<1\\ 0,otherwise \end{array}\right.$$

In addition, the corresponding high-pass filter is g[n]:

(26) $$g[n]=\left[\frac{1}{\sqrt{2}},-\frac{1}{\sqrt{2}}\right]$$

The Haar wavelet transform calculation steps are as follows: To begin with, we input the signal f[n]. Next, we calculate the discrete wavelet coefficients, where cA[k] is the approximation coefficient, which captures the low-frequency components of the signal and reflects the overall trend, providing a denoised trend baseline for the LSTM to reduce overfitting to noise; cD[k] is the detail coefficient, which captures the high-frequency components of the signal and reflects local changes, guiding the attention mechanism to focus on key change points.

(27) $$\left\{\begin{array}{l} cA_{j}[k]=\sum_{n}f[n]\cdot \phi_{1,k}[n]\\ \phi_{1,k}[n]=\frac{1}{\sqrt{2}}\phi (\frac{n}{2}-k)\\ cD_{j}[k]=\sum_{n}f[n]\cdot \varphi_{1,k}[n]\\ \varphi_{1,k}[n]=\frac{1}{\sqrt{2}}\varphi (\frac{n}{2}-k) \end{array}\right.$$

Finally, we verify the correctness of the decomposition through inverse transformation, and reconstruct the formula as follows:

(28) $$f_{reconstructed}[n]=\sum_{k}cA[k]\cdot \phi_{1,k}[n]+\sum_{k}cD[k]\cdot \varphi_{1,k}[n]$$

The multi-head attention mechanism is capable of simultaneously focusing on different parts of the input sequence by computing multiple attention heads in parallel, enabling the extraction of more comprehensive features. The calculation of the multi-head attention mechanism is mainly divided into the following steps:

Firstly, we project the input data separately onto Query (*Q*), Key (*K*), and Value (*V*).

(29) $$\left\{\begin{array}{l} Q=outputs\cdot W_{Q},W_{Q}\in R^{d_{\mathrm{mod}el}\times d_{k}\cdot h}\\ K=outputs\cdot W_{K},W_{K}\in R^{d_{\mathrm{mod}el}\times d_{k}\cdot h}\\ V=outputs\cdot W_{V},W_{V}\in R^{d_{\mathrm{mod}el}\times d_{V}\cdot h} \end{array}\right.$$

Then, we split *Q*, *K*, and *V* according to the number of heads:

(30) $$\left\{\begin{array}{l} Q\to [Q_{1},\ldots ,Q_{h}],Q\in R^{L\times d_{k}}\\ K\to [K_{1},\ldots ,K_{h}],K\in R^{L\times d_{k}}\\ V\to [V_{1},\ldots ,V_{h}],V\in R^{L\times dv} \end{array}\right.$$

Additionally, we use the scaled dot-product attention to calculate each head independently:

(31) $$head_{i}=soft\max(\frac{Q_{i}K_{i}^{T}}{\sqrt{d_{k}}})V_{i}$$

*Q* is the query matrix, *K* is the key matrix, *V* is the value matrix, and dk is the dimension of the key. *T* represents the time step length of the input sequence, and ${Q_{i}K_{i}}^{T}$ is a T×T matrix representing the similarity weights between all-time steps. *V* is the value matrix, carrying the feature information to be aggregated, and is the direct source of the attention output. By calculating the T×T eights through ${Q_{i}K_{i}}^{T}$, *V* is weighted and summed, ultimately outputting features of the same dimension as *V*.

Finally, concatenate the outputs of all heads and project them. We use $W^{O}$ to maintain consistent dimensions:

(32) $$MultiHeadAttention(Q,K,V)=Concat(head1,head2,\ldots ,headh)W^{O}$$

where headi represents the projection matrix of each attention head, and $W^{O}$ denotes the linear transformation matrix of the output. The multi-head attention mechanism effectively captures global dependencies within the input sequence, enhancing the model’s ability to manage long-range relationships. Additionally, residual connections are employed to retain the features of the original inputs, thereby preventing information loss.

**Figure 2:**
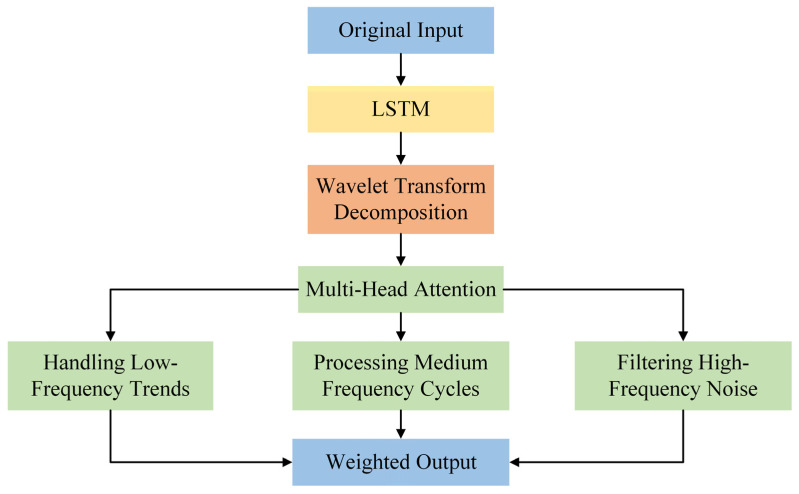
The schematic diagram of Wave-MH attention.

In the mWM Block, the wavelet transform, and the multi-head attention mechanism are integrated in several stages, as shown in Figure 2. First, the input data undergoes wavelet transforms to generate multi-scale wavelet coefficients, which are subsequently consolidated into a tensor and input into the LSTM block for time-series feature extraction. The output of the LSTM block is then processed through the multi-head attention mechanism to capture global dependencies, with residual connections preserving the original features.

The fusion of wavelet transforms and multi-head attention facilitates effective multi-scale feature extraction and global dependency modeling, addressing the challenges posed by uncertain events. This enables the model to dynamically capture both trends and cyclical patterns in carbon price data over time, thereby improving performance and prediction accuracy for non-stationary time series signals. Moreover, this integration not only enhances our understanding of the underlying mechanisms driving carbon prices but also allows for a comprehensive analysis of the complex dynamics of the carbon trading market. It further strengthens the model’s robustness to noise interference, ensuring stable predictions of carbon price fluctuations in non-stationary market conditions.

## 3. Result and Analysis

### 3.1. Experimental Environment and Training Details

To ensure the accuracy and reliability of the DKWM-XLSTM results, all experiments were conducted in a controlled hardware and software environment provided by the AutoDL platform. A standardized software development environment was utilized to maintain consistency in operating systems and tools. The hardware and software configurations are detailed in Table 6.

### 3.2. Evaluation Indicators

Robust performance indicators are crucial for validating the proposed prediction framework. In this study, we utilize three widely recognized categories of metrics: absolute error metrics, relative error metrics, and goodness-of-fit metrics.

(1) Absolute error metrics include:

The Mean Square Error (MSE) quantifies the average deviation between the predicted and actual carbon prices, providing an effective measure of model accuracy in capturing carbon market fluctuations. By analyzing MSE, it is possible to identify the primary sources of prediction errors, which can be addressed to optimize the model’s performance. This enhancement improves the model’s ability to forecast future carbon price trends, offering market participants a more reliable tool for decision-making. The MSE value is greater than or equal to 0, with smaller values indicating better model performance. The formula for this indicator is as follows:

(33) $$MSE=\frac{1}{n}\sum_{i=1}^{n}{\left(y_{i}-\hat{y_{i}}\right)}^{2}$$

The Mean Absolute Error (MAE) represents the overall magnitude of deviation in carbon price forecasts, providing policymakers and businesses with a clear measure of forecast accuracy. By analyzing the MAE, it is possible to assess the model’s performance across different time periods or market conditions, enabling the adjustment of carbon trading strategies and investment decisions to mitigate economic losses and risks. The MAE value ranges from 0 and above, with lower values indicating superior model performance. The formula for this indicator is as follows:

(34) $$MAE=\frac{1}{n}\sum_{i=1}^{n}\left|y_{i}-\hat{y_{i}}\right|$$

(2) Relative error metrics include:

The Mean Absolute Percentage Error (MAPE) quantifies the average proportion of prediction errors in relation to actual carbon prices, offering an effective measure of the model’s relative accuracy across different price levels. MAPE is particularly valuable for evaluating model performance in response to fluctuations in carbon market prices, providing market participants with a tool to assess risk and develop more adaptive trading strategies. The MAPE value ranges from 0% to 100%, with values closer to 0% indicating better model performance. The formula for this indicator is as follows:

(35) $$MAPE=\frac{100\%}{n}\sum_{i=1}^{n}\left|\frac{y_{i}-\hat{y_{i}}}{y_{i}}\right|$$

(3) Goodness-of-fit metrics:

The coefficient of determination (R^2^) is employed to quantify the explanatory power of carbon price prediction models in relation to market fluctuations, offering investors and policymakers an intuitive measure of model fit. A higher R^2^ value signifies that the model is more effective in capturing trends and variations in the carbon market. This enables users to assess the model’s reliability and make more informed decisions in carbon trading and policy formulation. The R^2^ value ranges from 0% to 100%, with values approaching 100% being more desirable. The formula for this indicator is as follows:

(36) $$R^{2}=1-\frac{\sum_{i=1}^{n}{\left(y_{i}-\hat{y_{i}}\right)}^{2}}{\sum_{i=1}^{n}{\left(y_{i}-\bar{y}\right)}^{2}}$$

### 3.3. Ablation Experiments

To ensure fairness and network stability, we conducted ablation experiments on three key modules: the DECOMP data decomposition module, the KAN-MD feature extraction module, and the Wave-MH attention module. Under controlled experimental conditions, we performed 16 experiments, and the results are presented in Table 7, accompanied by detailed analysis and comparison.

In this study, the DECOMP module addresses the challenge of carbon price fluctuations influenced by multiple non-stationary factors. By integrating data decomposition with XLSTM, this module significantly enhances the predictability of the input data, thereby establishing a solid foundation for subsequent feature extraction. Experimental results indicate that the inclusion of the DECOMP module reduced the MAE by 0.0007 and the MSE by 0.041%. This module improves data predictability and overall model performance by decomposing data into trend and cyclical features and inputting them into separate channels via a dual-channel approach.

The KAN-MD module optimizes feature extraction by effectively reducing the interference of redundant information, thereby significantly enhancing the accuracy of trend-based and periodic feature extraction. Compared to the baseline model, the KAN-MD module reduced the MAE by 0.0006 and the MSE by 0.043%, demonstrating exceptional feature extraction capabilities and robustness, particularly in managing redundant information.

The Wave-MH attention module integrates wavelet transformation with a multi-head attention mechanism to better account for the impact of uncertain events, enhancing training stability. Experimental results show that after the application of the Wave-MH attention module, the MSE decreased by 0.039%, and the MAE decreased by 0.0018. The Wave-MH attention mechanism enables the model to respond more effectively to dynamic market changes, thereby improving overall model stability.

The combined performance of these three modules is outstanding, with the model achieving an MSE of 0.184%, an RMSE of 9.07, an MAE of 0.0244, and an R^2^ of 96.06%. These results demonstrate the model’s robust capabilities in time series forecasting.

### 3.4. Comparison Experiments with Other Networks

To validate the proposed method, four commonly used network models—Backpropagation (BP) [42], Temporal Convolutional Network (TCN) [43], Gated Recurrent Unit (GRU) [44], and Bidirectional Long Short-Term Memory (Bi-LSTM) [17], along with Transformer [45] and Prophet [46]—were selected as baseline models for comparison. A comprehensive performance analysis was then conducted, with the results presented in Table 8. The accuracy of the proposed network significantly outperforms the traditional BP model. Specifically, the MAE of the BP model is 0.0283. The BP neural network lacks the ability to retain event sequence data, thereby failing to effectively capture temporal dependencies. As the data volume increases, the BP network is prone to overfitting. TCN demonstrates strong performance in processing long sequences and excels in feature extraction. However, its effectiveness can be influenced by variations in the time series order, leading to fluctuations in model performance and hindering reliable feature extraction. The MSE for GRU is 0.204%. Although GRU has a simpler structure than XLSTM, it tends to lose memory of earlier information when processing long time series. Bi-LSTM, on the other hand, can simultaneously learn from both past and future data, improving time series prediction accuracy. However, Bi-LSTM is prone to overfitting in the presence of insufficient data, which compromises its generalization ability. The MAE of the Transformer model is 0.095. Its core advantage lies in the self-attention mechanism, which can efficiently model long-term dependencies in time-series data and supports parallel computing and multi-variable inputs, making it particularly suitable for complex prediction scenarios involving large amounts of data. However, due to the late development of China’s carbon market, which is currently in the pilot expansion phase, the available market data is relatively limited. This limits the Transformer model’s ability to leverage its reliance on large-scale data, potentially resulting in performance that may not match that of other models in this specific scenario. The MSE of the Prophet is 5.48%, which is relatively poor. This may be related to its statistical modeling method, as Prophet relies on segmented linear trends and fixed seasonal patterns. It has weak adaptability to sudden fluctuations and is unable to capture the complex relationship between carbon prices and multidimensional factors, resulting in poor performance in carbon price forecasting.

In contrast, the proposed method demonstrates exceptional performance in carbon price time series forecasting. The DECOMP module effectively separates the data into trend and cyclical features, mitigating the interference of non-stationary multi-factors and facilitating more accurate feature extraction. The KAN-MD module enhances feature extraction by rapidly eliminating redundant information, while the Wave-MH attention mechanism captures multi-scale features and global relational dependencies within the carbon price data, thus improving the robustness of the overall model. These modules work synergistically to significantly enhance the model’s ability to manage complex environments, ensuring stable training and high accuracy.

**Figure 3:**
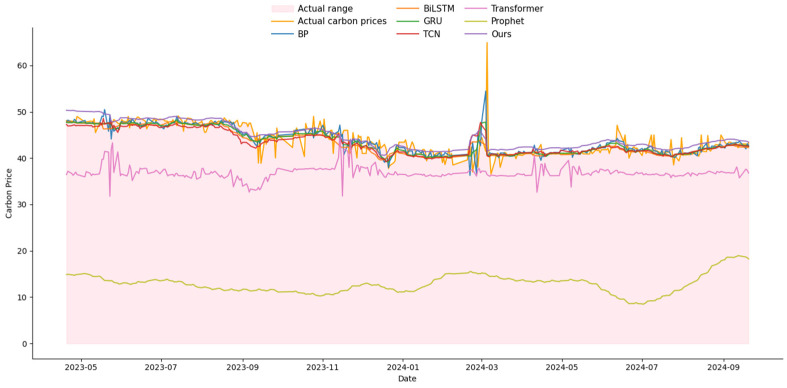
The schematic diagram of multi-head attention (Test_Size = 0.2).

**Figure 4:**
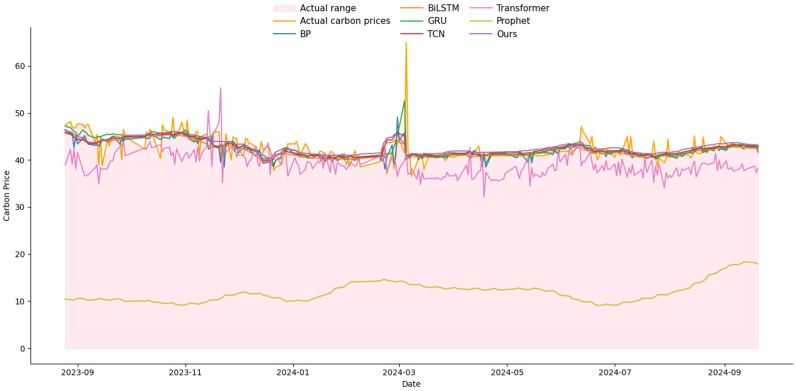
The schematic diagram of multi-head attention (Test_Size = 0.15).

The comprehensive analysis reveals that the three modules in this method significantly improve the model’s feature extraction capability, leading to enhanced prediction accuracy. As a result, this method presents a reliable solution for forecasting carbon prices in a time series of tasks. The MSE of the proposed method is 0.204%, the MAE is 0.0277, the MAPE is 9.25%, and the R^2^ value is 96.06%. All performance metrics notably surpass those of the other models. Detailed results are presented in Table 8. Moreover, a comparative analysis between predicted and actual values across all networks is presented in Figure 3 and Figure 4, demonstrating the superior predictive capability of our method.

### 3.5. Hyperparameter Optimization Experiments

This section evaluates the quantitative impact of key parameters, such as the hidden layer dimension in the DKWM-XLSTM model, on carbon price prediction performance through a series of hyperparameter tuning experiments. The findings provide an optimization strategy for key parameters in constructing a carbon price analysis framework that balances physical interpretability and predictive accuracy. Additionally, the methodology serves as a general reference for modeling complex systems in energy finance.

An experiment was conducted comparing the prediction performance of single-layer and double-layer LSTM structures. The detailed results are presented in Table 9. The results show that the MSE decreases from 0.215% to 0.209%, representing a 2.8% reduction with the two-layer structure. This suggests that incorporating additional network layers enhances the model’s ability to detect complex patterns in the time series. However, the robustness of the deeper network declines, as reflected in the increase in MAPE from 9.03% to 12.03%. This indicates that the deeper model is more susceptible to overfitting the specific fluctuations present in the training data when abnormal variations occur. Notably, the R^2^ value for the two-layer structure improves to 0.9611, demonstrating the model’s enhanced capability to accurately capture data patterns. Based on an analysis of four core metrics—MSE, MAE, MAPE, and R^2^ (*p* < 0.05, no significant overlap in confidence intervals)—the single-layer LSTM performed better in terms of prediction stability, computational efficiency, and generalization ability, so this structure was ultimately selected.

Furthermore, an additional experiment was conducted to compare the prediction performance across four different hidden layer configurations. The results show that the configuration of [8, 16] achieves the fastest training time while maintaining the lowest MSE of 0.00200. However, when the hidden layer was expanded to [64, 128], the MSE increased by 5.5% (reaching 0.00211), and the training time increased by more than three times. These findings suggest that excessively large hidden layers may negatively affect model performance, with prediction accuracy starting to decline once the total number of neurons exceeds twice the dimension of the input features (12 dimensions in this experiment). The detailed results are presented in Table 10.

## 4. Discussion

This network integrates the DECOMP (data decomposition) module, the KAN-MD feature extraction module, and the Wave-MH attention mechanism; they are specifically designed to address the complexities of carbon trading prices, which are influenced by multiple interrelated factors. These challenges include difficulties in feature extraction and the non-stationary nature of market dynamics.

The prediction outcomes of this model surpass those of competing networks; however, several limitations remain. (1) The method’s intricate design, which combines the XLSTM network with three additional modules, results in a larger model size and slower operational speed. It is important to acknowledge that the reduced processing speed may not be solely attributed to the architectural complexity; factors such as data preprocessing techniques and hyperparameter configurations during training could also influence overall processing time. (2) The training dataset predominantly consists of factors related to carbon trading prices in Hubei, which may introduce bias and limit the model’s applicability to other agricultural products or market conditions. The dynamics of Hubei’s carbon prices may not fully represent other carbon markets. Furthermore, data preprocessing methods, including normalization and augmentation, can impact model performance; if these techniques are not specifically tailored to the characteristics of different sectors, they may hinder the model’s effectiveness. (3) The Chinese carbon market encompasses eight regions, including Hubei, Beijing, Tianjin, Chongqing, Guangzhou, Fujian, Shanghai, and Shenzhen, each serving distinct industries and exhibiting varying levels of development. Some markets experience low trading volumes and are subject to diverse external influences. The performance of this model in these alternative markets requires further investigation.

Additionally, to enhance the robustness and applicability of this model, several other considerations should be addressed:

(1) Data quality requirements: Time-series forecasting imposes stringent demands on both the quality and quantity of the dataset. Certain carbon trading markets in China suffer from limited effective transaction data. Therefore, further optimization of this method is necessary to facilitate its application across diverse carbon trading markets.

(2) Related influencing factors: In real-world carbon trading markets, carbon prices are influenced by a broader range of factors than those included in the current model, such as environmental changes and external variables. However, due to the challenges associated with data collection and the inherent uncertainty surrounding these factors, they are not fully represented in the current dataset. Future research should aim to incorporate a more comprehensive set of relevant influencing factors to improve the model’s practicality and robustness, ensuring that it better reflects real-world conditions.

(3) Generalizability of the Method: While the model has demonstrated promising results in forecasting carbon prices within the Hubei carbon market, further testing is required to fully assess its generalizability to other national carbon markets (e.g., South Korea, New Zealand). Although the model has exhibited strong performance, its adaptability to different carbon markets and other financial derivatives remains to be comprehensively evaluated. To ensure the method’s effective application in real-world carbon trading scenarios, it is crucial to expand the dataset to incorporate a wider range of influencing factors. This expansion will help validate the model’s performance and ensure its reliability and robustness across diverse carbon markets and a broader spectrum of influencing factors. Additionally, further testing with actual carbon price data will provide valuable insights into optimizing the model for improved performance under varying conditions in real carbon markets.

Future Research Directions: Future investigations will focus on the following objectives:

(1) Enhancing the scale and parameters of the method to improve the accuracy and precision of sales forecasts.

(2) Expanding the dataset to incorporate additional highly relevant influencing factors, such as environmental monitoring data provided by remote sensing technology, to further augment the model’s learning capabilities.

(3) When establishing datasets for other carbon trading markets, it is essential to include factors specific to those markets to address discrepancies in carbon market forecasting results across diverse environments.

The overarching goal is to develop an efficient and stable carbon trading price prediction model that effectively decomposes data and adapts to the complexities of market dynamics. This model will provide reliable technical support for pricing carbon financial derivatives and managing associated risks.

## 5. Conclusions

Accurate forecasting of carbon trading prices is critical for establishing a multi-source, heterogeneous data integration mechanism in carbon markets and regulating forest carbon sink projects. This technology aids investors in making informed decisions and provides reliable technical support for pricing carbon financial derivatives and managing risk. Advanced forecasting methods can improve management efficiency, reduce risk uncertainty, and foster the development of China’s carbon market. However, fluctuations in carbon trading prices are closely linked to various factors, including seasonal influences, market dynamics, and government policies, which complicate forecasting efforts.

This study introduces the DKWM-XLSTM model, a novel approach designed to address these challenges. The innovation of this method lies in its integration of decomposition techniques with the XLSTM dual-channel framework, thereby enhancing data feature extraction capabilities. The DECOMP module decomposes the data into trend-based and cyclical components, which are then fed into the sKAN and mWM blocks of the XLSTM module to further improve feature extraction while accounting for multiple relevant factors. The KAN-MD module optimizes feature extraction by reducing redundant information, while the Wave-MH attention module enhances the model’s ability to focus on multi-scale features. This, in turn, improves the model’s adaptability to dynamic changes in the carbon market, enhancing both convergence and robustness. The results of ablation experiments demonstrate that these modules significantly improve model performance, with reductions in MSE, MAPE, and MAE by 6.7%, 9.6%, and 12.8%, respectively, while R^2^ increased by 0.6%. Compared to the Bi-LSTM model, our method achieves reductions of 3.14% in MSE, 2.39% in MAPE, and 3.78% in MAE, while also increasing R^2^ by 0.13%. These results further validate the superior prediction accuracy of our method.

The proposed method outperforms several prominent time-series prediction networks across multiple performance metrics, achieving an MSE of 0.204% and an MAE of 0.0277%. The method effectively addresses complex factors, extracts features with high efficiency, and reduces redundant information, demonstrating its potential for broad application in the carbon price market. These improvements offer the prospect of more accurate and rational investment decisions, thereby supporting the sustainable development of China’s carbon market.

Looking ahead, the applicability of this methodology can be extended to other financial derivatives, such as predicting price trends in various carbon markets. Future research will focus on incorporating additional highly relevant influencing factors into the dataset to further enhance the accuracy of model predictions. Moreover, datasets encompassing trading prices from other carbon markets, along with corresponding influencing factors, will be developed to ensure the method’s versatility across different markets. Furthermore, integrating remote sensing data into the analysis will provide a more precise assessment of carbon sink potential across various regions. With these advancements, this methodology is poised to significantly contribute to the ongoing enhancement of China’s carbon trading market, risk management practices, and the support of sustainable development for forestry carbon sinks.

## Figures and Tables

**Table 1:** Classification and interpretation of variables in datasets.

Category	Code	Symbol	Explanation
Energy	Y	Carbon Price	Price per ton of CO_2_ equivalent in carbon markets
	X1	Market Price of Liquefied Natural Gas	Market price of liquefied natural gas
	X2	Gasoline Price	Per-unit price of gasoline in markets
	X3	Diesel Price	Per-unit price of diesel in markets
Economy	X4	Gross Domestic Product (GDP)	Total economic output of a country
	X5	Manufacturing Purchasing Managers’ Index (PMI)	Indicating sector expansion or contraction
	X6	Producer Price Index (PPI)	Tracking output price changes in industries
Society	X7	Consumer Price Index (CPI)	Measuring price changes of consumer goods or services
	X8	Inflation Rate	Percentage increase in general price level

**Table 2:** Descriptive statistics of key variables.

Code	N	MEAN	SD	MIN	MEDIAN	MAX
lnY	1853	3.4361	0.4398	2.4423	3.4648	10.6712
lnX1	1853	8.4617	0.2751	7.8633	8.4338	9.1160
lnX2	1853	9.0822	0.1236	8.8298	9.0797	9.3312
lnX3	1853	8.9602	0.1334	8.6844	8.9625	9.2292
X4	1659	0.3098	0.8948	−2.3026	0.5878	1.6864
lnX5	1853	4.6208	0.0123	4.5971	4.6214	4.6578
lnX6	1853	4.6240	0.0437	4.5497	4.6092	4.7318
lnX7	1853	3.9162	0.0379	3.5752	3.9160	3.9627
lnX8	1853	11.6529	0.7817	10.0894	11.8357	12.7861

**Table 3:** Results of correlation analysis among variables.

Code	lnY	lnX1	lnX2	lnX3	X4	lnX5	lnX6	lnX7	lnX8
lnY	1.0000								
lnX1	0.3820 ***	1.0000							
lnX2	0.5400 ***	0.6339 ***	1.0000						
lnX3	0.6282 ***	0.5808 ***	0.9009 ***	1.0000					
X4	−0.2652 ***	−0.0960 ***	−0.2414 ***	−0.2066 ***	1.0000				
lnX5	−0.2360 ***	−0.1718 ***	−0.2271 ***	−0.1697 ***	0.8802 ***	1.0000			
lnX6	−0.2717 ***	0.2876 ***	0.0536 **	−0.0513 **	0.1645 ***	0.1024 ***	1.0000		
lnX7	−0.3374 ***	−0.1541 ***	−0.2511 ***	−0.3364 ***	−0.1038 ***	−0.1891 ***	0.1715 ***	1.0000	
lnX8	0.8167 ***	0.3376 ***	0.4914 ***	0.5753 ***	−0.4570 ***	−0.4115 ***	−0.3942 ***	−0.2990 ***	1.0000

Note: *, **, and *** indicate significance at 10%, 5%, and 1% significance levels, respectively (two-tailed test).

**Table 4:** Results of augmented Dickey–Fuller test for key variables.

Code	ADF Value	1% Critical Value	5% Critical Value	10% Critical Value	Conclusion
lnY	−2.099	−3.430	−2.860	−2.570	Non-Stationary
d.lnY	−74.126	−3.430	−2.860	−2.570	Stationary
lnX1	−3.113	−3.430	−2.860	−2.570	Stationary
d.lnX1	−43.002	−3.430	−2.860	−2.570	Stationary
lnX2	−1.631	−3.430	−2.860	−2.570	Non-Stationary
d.lnX2	−43.003	−3.430	−2.860	−2.570	Stationary
lnX3	−2.059	−3.430	−2.860	−2.570	Non-Stationary
d.lnX3	−43.010	−3.430	−2.860	−2.570	Stationary
X4	−1.800	−3.430	−2.860	−2.570	Non-Stationary
d.X4	−40.597	−3.430	−2.860	−2.570	Stationary
lnX5	−1.992	−3.430	−2.860	−2.570	Non-Stationary
d.lnX5	−43.005	−3.430	−2.860	−2.570	Stationary
lnX6	−1.044	−3.430	−2.860	−2.570	Non-Stationary
d.lnX6	−43.026	−3.430	−2.860	−2.570	Stationary
lnX7	−7.076	−3.430	−2.860	−2.570	Stationary
d.lnX7	−43.000	−3.430	−2.860	−2.570	Stationary
lnX8	−1.484	−3.430	−2.860	−2.570	Non-Stationary
d.lnX8	−43.128	−3.430	−2.860	−2.570	Stationary

**Table 5:** Comparative analysis of wavelet basis functions.

Training Set Ratio	Indicator	Haar	Meyer	Daubechies
80%	MSE	0.231%	0.240%	0.240%
	MAE	0.032	0.032	0.032
	MAPE	10.14	10.71	11.82
	R2	95.36%	95.35%	95.53%
85%	MSE	0.279%	0.250%	0.250%
	MAE	0.031	0.033	0.032
	MAPE	10.17	11.37	10.78
	R2	94.57%	95.14%	95.12%

**Table 6:** System configuration parameters.

	Designation	Version
Hardware	CPU	Intel Xeon Platinum 8474C
	RAM	80 GB
	GPU	NVIDIA GeForce RTX 4090D (24GB)
	Hard disk	System disk:30 GB Data disk:50 GB
Software	OS	Windows 11 ×64
	CUDA	11.1
	CUDNN	8.0.5
	Python	3.11.9
	Pytorch	1.8.1
	Tensorflow	2.18.0

**Table 7:** Results of ablation experiments.

Training Set Ratio	Group	DECOMP	KAN-MD	WAVE -MH Attention	MSE	RMSE	MAE	R^2^
80%	①	--	--	--	0.237%	10.04	0.0280	93.49%
	②	√			0.196%	9.34	0.0273	96.21%
	③		√		0.194%	9.37	0.0274	95.22%
	④			√	0.198%	9.42	0.0262	95.87%
	⑤	√	√		0.193%	9.26	0.0269	96.20%
	⑥		√	√	0.188%	9.21	0.0277	96.81%
	⑦	√		√	0.185%	9.18	0.0266	95.47%
	⑧	√	√	√	0.184%	9.07	0.0244	96.06%
85%	①	--	--	--	0.230%	11.12	0.0289	92.53%
	②	√			0.213%	9.41	0.0269	87.85%
	③		√		0.224%	9.62	0.0282	91.12%
	④			√	0.219%	9.47	0.0273	89.64%
	⑤	√	√		0.225%	13.12	0.0304	95.61%
	⑥		√	√	0.208%	12.80	0.0277	93.61%
	⑦	√		√	0.214%	11.72	0.0340	94.46%
	⑧	√	√	√	0.218%	11.05	0.0290	95.75%

Note: --: Inactive status; √: Active status.

**Table 8:** Result of comparison experiments with other networks.

Training Set Ratio	Indicator	BP	TCN	Bi-LSTM	GRU	Transformer	Prophet	Ours
80%	MSE	0.213%	0.208%	0.211%	0.214%	1.34%	5.48%	0.204%
	MAE	0.0283	0.0279	0.0284	0.0271	0.095	0.202	0.0277
	MAPE	9.52	9.59	9.62	9.26	31.14	128.79	9.25
	R^2^	95.28%	95.27%	94.93%	93.05%	74.01%	−6.04%	96.06%
85%	MSE	0.250%	0.233%	0.253%	0.217%	1.43%	5.47%	0.218%
	MAE	0.0301	0.0313	0.0304	0.0274	0.095	0.202	0.0290
	MAPE	8.96	10.11	9.26	8.96	39.77	134.52	11.05
	R^2^	93.13%	92.47%	92.08%	93.77%	72.19%	−6.64%	95.75%

**Table 9:** The number of layers of LSTM.

Size	MSE	MAE	MAPE	R^2^
One	0.215% [0.190%, 0.432%]	0.0287 [0.0229, 0.0413]	9.03 [8.81, 13.83]	95.83% [91.83%, 96.69%]
Two	0.209% [0.207%, 0.513%]	0.0288 [0.0223, 0.0485]	12.03 [11.23, 19.58]	96.11% [90.18%, 94.89%]

**Table 10:** Hidden layer size for LSTM.

Size	MSE	MAE	MAPE	R^2^
[8, 16]	0.200%	0.0288	12.03	96.11%
[16, 32]	0.202%	0.0287	11.04	96.09%
[32, 64]	0.204%	0.0275	10.16	96.05%
[64, 128]	0.211%	0.0279	9.32	95.92%

## Data Availability

Table 1 details the data used in this study, which originates exclusively from publicly accessible sources: the WIND Database and the official websites (https://www.hbets.cn/, accessed on 13 July 2025). Requests for raw data should be directed to the author, Yunlong Yu, at 20225793@csuft.edu.cn.

## Abbreviations and Nomenclature

**Abbreviation**	**Definition**
DECOMP	Decomposition
KAN-MD	Kolmogorov–Arnold Networks With Multi-Domain Diffusion
Wave-MH Attention	Wave-Multi-Head Attention
DKWM-XLSTM	Enhancing XLSTM With Decomposition, Kan-MD, And Wave-MH Attention Mechanisms
LSTM-CNN	Long Short-Term Memory and Convolutional Neural Network
GANs	Generative Adversarial Networks
ARIMA	Autoregressive Integrated Moving Average
ET-MVMD-LSTM	Extreme Random Trees and Multivariate Variational Modal Decomposition to Enhance Long Short-Term Memory
MEPT	Multiple Ensemble Patch Transformation
ICEEMDAN	Improved Adaptive Noise-Complete Ensemble Empirical Mode Decomposition
CTCNs	Causal Time Convolutional Networks
MIDE	Multi-Scale Interval Value Decomposition
NAMEMD	Noise-Assisted Multivariate Empirical Mode Decomposition
IVAR	Interval Value Vector Autoregression
IEA	Interval Event Analysis
IMLP	Interval Multi-Layer Perceptron
CEEMDAN	Complete Ensemble Empirical Mode Decomposition With Adaptive Noise
VMD	Variational Mode Decomposition
XGBoost	Extreme Gradient Boosting
PACF	Partial Autocorrelation Function
XLSTM	Extended Long Short-Term Memory
ADF	Augmented Dickey–Fuller
MLPs	Multi-Layer Perceptrons
CWT	Continuous Wavelet Transform
DWT	Discrete Wavelet Transform
MSE	Mean Squared Error
MAE	Mean Absolute Error
MAPE	Mean Absolute Percentage Error
R^2^	Coefficient Of Determination
BP	Backpropagation
TCN	Temporal Convolutional Network
GRU	Gated Recurrent Unit
Bi-LSTM	Bidirectional Long Short-Term Memory
Transformer	Transformer Architecture
Propht	Prophet Forecasting Model
**Nomenclature**	**Definition**
Variables	
Y	Carbon Price
X1	Market price of liquefied natural gas
X2	Gasoline Price
X3	Diesel Price
X4	Gross Domestic Product (GDP)
X5	Manufacturing Purchasing Managers’ Index (PMI)
X6	Producer Price Index (PPI)
X7	Consumer Price Index (CPI)
X8	Inflation Rate
Parameters	
$x_{t}$	Total term of dataset
$T_{t}$	Trend term of dataset
$S_{t}$	Cyclical term of dataset
m	The dynamic half-window width
P	The dominant cycle length
k	The neighborhood offset relative to the current calculation point t
$f_{t}$	Forgetting gate of the mWM block
$i_{t}$	Input gate of the mWM block
$o_{t}$	Output gate of the mWM block
$C_{t}$	Cell state of the mWM block
$h_{t}^{M}$	Hidden state of the mWM block
${f^{\prime }}_{t}$	The forgetting gate of the sKAN block
${i^{\prime }}_{t}$	The input gate of the sKAN block
${o^{\prime }}_{t}$	The output gate of the sKAN block
${C^{\prime }}_{t}$	The cell state of the sKAN block
$h_{t}^{s}$	The hidden state of the sKAN block
$h_{t}$	Combine the mWM block and sKAN block hidden states to generate the prediction results
$\hat{Y_{t}}$	The final prediction result
$W_{p}$	The weights of the fully connected layer
$b_{p}$	The biases of the fully connected layer
W	The weights of KAN-MD
b	The biases of KAN-MD
c	The number of layers of KAN-MD
$Z_{input}$	The layer of KAN-MD
ReLU	The activation function of KAN-MD
N	The number of samples
$y_{true}^{\left(i\right)}$	The true value of the i-th sample
$y_{pred}^{\left(i\right)}$	The predicted value of the i-th sample
θ	The parameters of KAN-MD
$\theta_{t}$	The model parameter at the t-th iteration
η	The learning rate of loss function
∇θ⋅J(θ)	The gradient of the loss function J(θ) with respect to the parameter θ
f[n]	The input signal of wavelet transform
$\varphi_{a,b}(t)$	The wavelet basis function
a	The scale parameter of wavelet transform
b	The shift parameter of wavelet transform
ϕ(t)	The scale function of Haar wavelet
h[n]	Low-pass filter of scale function
ψ(t)	The wavelet function of Haar wavelet
g[n]	High-pass filter of wavelet function
$cA_{j}[k]$	The approximation coefficient
$cD_{j}[k]$	The detail coefficient
k	Translation parameter
j	Number of scales
Q	Query matrix
K	Key matrix
V	Value matrix
T	The time step length of the input sequence
$W^{q}$	Query weight matrix
$W_{k}$	Key weight matrix
$W_{v}$	Value weight matrix
$W^{o}$	The linear transformation matrix of the output
$head_{i}$	The projection matrix of each attention head
